# Integrated Multi-Omics Approaches Provide Novel Insights into the Mechanisms Underlying Signature Flavor Development in Mulberry Fruits

**DOI:** 10.3390/foods14193309

**Published:** 2025-09-24

**Authors:** Jiamei He, Xing Zhang, Song Chen, Jiahu Yang, Zhengang Li

**Affiliations:** 1 Sericulture and Apiculture Research Institute, Yunnan Academy of Agricultural Sciences, Mengzi 661101, Chinaynchensong@163.com (S.C.); csyangjiahu26@126.com (J.Y.); 2 College of Agronomy and Biotechnology, Yunnan Agricultural University, Kunming 650201, China; bryan960614@163.com

**Keywords:** *Morus laevigata*, flavor, fatty acid metabolites, flavonoid metabolites, oxidation, nutritional balance

## Abstract

With the increasing consumption of mulberry fruits in commercial markets, flavor profiles have emerged as critical determinants of consumer preference and market acceptance. This investigation utilized four *Morus laevigata* (*Morus* L.) accessions exhibiting pronounced variations in fruit pigmentation and flavor characteristics as experimental materials. Comprehensive two-dimensional gas chromatography coupled with time-of-flight mass spectrometry (GC × GC-TOF MS) was employed to identify key volatile aromatic compounds, while integrated untargeted metabolomics and transcriptomics approaches were applied to elucidate the underlying mechanisms of flavor biosynthesis. Analysis revealed that aldehydes, ketones, lactones, and heterocyclic compounds constitute the primary volatile organic compounds responsible for *M. laevigata* flavor complexity. The biosynthesis of these volatile aromatic compounds exhibits a direct correlation with lipid metabolite oxidation pathways. Concurrently, oxidative processes are modulated by *M. laevigata* flavonoid metabolites with antioxidant properties, which subsequently regulate both the compositional profile and quantitative distribution of volatile aromatic compounds. These findings offer novel insights into the metabolite–volatile compound interactions within mulberry systems, establishing a foundational framework for advancing fruit flavor research and cultivar development programs.

## 1. Introduction

Mulberry trees (*Morus* spp.) represent a versatile species within the *Moraceae* family, demonstrating significant applications across livestock management, healthcare, and industrial sectors [1]. These trees exhibit substantial ecological value through their root systems, which enhance soil water retention capacity while generating exudates that enrich and expand soil microbial communities [2]. Furthermore, the aerial biomass facilitates atmospheric pollutant absorption, while the root system enables soil contaminant uptake, establishing mulberry trees as effective carbon sequestration agents and phytoremediation candidates for heavy metal contamination [3,4]. Additionally, mulberry fruits contain abundant proteins, minerals, vitamins, amino acids, anthocyanins, alkaloids, and diverse bioactive compounds, supporting their extensive utilization in therapeutic and nutraceutical applications [5,6]. The distinctive organoleptic properties and health-promoting attributes of mulberries have contributed to their increasing adoption as functional food products [7].

Mulberry flavor profiles result from the complex interplay between aromatic and gustatory components. Gustatory perception is primarily attributed to non-volatile compounds, with soluble sugars including sucrose and glucose, alongside organic acids such as citric acid, serving as the principal determinants [8]. Aromatic characteristics are governed by volatile organic compounds. These volatile compounds encompass several chemical classes, notably aldehydes, esters, alcohols, and terpenes [9]. Comprehensive investigations of mulberry flavor have incorporated multiple variables, including cultivar differences, environmental conditions, developmental stages, and both pre-harvest and post-harvest processing parameters [10,11].

The contribution of volatile organic compounds to aromatic profiles is determined not solely by their concentration levels, but also by their respective olfactory threshold values [12]. Consequently, odor activity values (ROAVs) serve as the standard metric for evaluating the integrated influence of both concentration and threshold parameters on overall aroma perception [13]. Contemporary research demonstrates that flavor compound biosynthesis and regulation across numerous plant species primarily involves three major metabolic pathways: fatty acid metabolism [14], the shikimic acid pathway [15], and terpenoid biosynthesis [16], with critical flavor precursors and associated regulatory genes being systematically characterized. For instance, in peach (*Prunus persica* L. Batsch), the alcohol acyltransferase gene *PpAAT1* undergoes transcriptional activation by PpNAC1, thereby facilitating ester biosynthesis [17]. In strawberry (*Fragaria* spp.), essential transcription factors FaDOF2 and FaMYB10, along with their downstream target genes *FaEGS2* and *FaEOBII*, have been characterized as regulators of phenylpropanoid volatile compound synthesis, particularly eugenol production, with subsequent elucidation of their coordinated regulatory networks [18]. Furthermore, transcription factors PuWRKY24, PuIAA29, and PuTINY have been implicated in aroma development during fruit maturation in ‘Nanguo’ pear (*Pyrus ussuriensis* Maxim.) [19]. Flavor biosynthesis represents a multifaceted process encompassing the entire cascade from gene expression to metabolite accumulation [14], including competitive interactions for shared substrates among distinct biosynthetic pathways [20]. These metabolite interactions collectively influence the enzymatic conversion of flavor precursors into volatile compounds that define the final aromatic profile [21]. Nevertheless, compared to other commercially important fruit species, investigations into the molecular mechanisms governing mulberry fruit flavor development remain limited, resulting in a paucity of research characterizing premium mulberry flavor attributes and their underlying regulatory mechanisms.

Fruit pigmentation represents an additional critical phenotypic characteristic influencing consumer preferences, primarily determined by flavonoid biosynthetic pathways. These flavonoid compounds demonstrate diverse bioactive properties, including antioxidant capacity, anti-inflammatory activity, immunomodulatory functions, and antimicrobial efficacy [22,23]. Prior research has demonstrated that red-fleshed peach fruits exhibit significantly lower levels of volatile compounds relative to their white-fleshed counterparts [24]. Analogous reductions in key aroma-contributing volatile compounds have also been reported in red-fleshed apple and grape cultivars [25,26]. The relationship between metabolite composition and flavor profiles in mulberry fruits presents significant research opportunities for comprehensive investigation.

Contemporary omics technologies have established robust analytical frameworks for elucidating the molecular mechanisms underlying fruit quality development. The integration of transcriptomic and metabolomic analyses represents a sophisticated approach for identifying critical regulatory genes, characterizing metabolic networks, and deciphering gene–metabolite interactions across diverse plant species [27]. For instance, integrated transcriptomic and metabolomic profiling has identified putative regulatory genes governing terpene biosynthetic pathways in tea (*Camellia sinensis*) [28]. Integrated metabolomic and transcriptomic analyses have elucidated the regulatory mechanisms governing flavonoid biosynthesis in purple tea cultivars [29]. In watermelon research, integrated omics methodologies have delineated comprehensive metabolite accumulation patterns and transcriptional regulatory networks throughout fruit ontogeny in both wild and domesticated genotypes [30]. Mulberry research has similarly employed integrated transcriptomic and metabolomic approaches, with temporal analyses revealing dynamic metabolite and transcript profiles throughout fruit development and ripening processes [31]. Specifically, comprehensive transcriptomic and metabolomic investigations of the ‘Baichang’ cultivar (*Morus macroura*) have characterized the molecular determinants governing creamy flavor development [32]. Advances in flavoromics technologies have enabled comprehensive characterization of food flavor profiles. GC × GC-TOF MS has emerged as a predominant analytical platform, offering superior throughput, precision, sensitivity, temporal resolution, and comprehensive qualitative capacity [33]. This methodology demonstrates enhanced volatile compound detection capabilities relative to conventional GC-MS and has been extensively implemented across diverse food matrices, including fruits and coffee, establishing itself as a cornerstone technology in flavor analysis [34,35]. Despite the demonstrated analytical superiority of GC × GC-TOF MS in fruit and vegetable flavor characterization, its application to mulberry volatile profiling remains markedly limited. Consequently, integrating transcriptomic, metabolomic, and volatolomic approaches represents a critical research imperative for systematically elucidating the genetic determinants, metabolic networks, and regulatory mechanisms governing mulberry flavor development, thereby advancing the field to unprecedented analytical depths.

This investigation employed four *M. laevigata* germplasm accessions from Yunnan Province, characterized by distinctive fruit color and flavor phenotypes. Through integrated volatile flavoromics, untargeted metabolomics, and transcriptomic analyses, we characterized the metabolic and gene expression profiles of *M. laevigata* to identify critical volatile compounds, examine gene–metabolite associations and metabolite–metabolite interactions, and elucidate the regulatory networks governing volatile flavor compound biosynthesis. These findings provide comprehensive insights into flavor-associated metabolic pathways and regulatory processes in mulberry fruits, offering valuable guidance for flavor-targeted breeding programs aimed at enhancing fruit quality.

## 2. Materials and Methods

### 2.1. Materials

**Figure 1:**
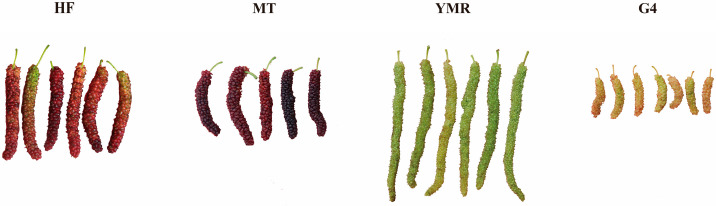
Mature fruits of each *M. laevigata* harvested 40 days post-pollination.

This investigation utilized four wild *M. laevigata* germplasm accessions maintained at the Yunnan Provincial Resource Repository of the Sericulture and Apiculture Research Institute, Yunnan Academy of Agricultural Sciences (Mengzi, Yunnan, China; 23.51° N, 103.40° E). The four *M. laevigata* exhibited distinct fruit coloration at maturity: ‘HF’ (HongFeng) displayed red fruits, ‘MT’ (MiTao) exhibited purple-red fruits, ‘YMR’ (YuMeiRen) presented yellow fruits, and ‘G4’ (GuanShang4) demonstrated yellow-green coloration (Figure 1). Fifteen individuals of each *M. laevigata* were cultivated, and mature fruits were harvested 40 days post-pollination. Sampling employed three biological replicates per *M. laevigata*, with each replicate comprising pooled mature fruits from three individual trees. Samples were immediately flash-frozen in liquid nitrogen and subsequently stored at −80 °C.

### 2.2. Measurement of Volatile Flavor Compounds in M. laevigata

Volatile compound analysis of samples ‘HF’, ‘MT’, ‘YMR’, and ‘G4’ was performed using comprehensive two-dimensional gas chromatography time-of-flight mass spectrometry (GC × GC-TOF-MS, Model 7890A, Agilent Technologies Inc., Santa Clara, CA, USA). Sample preparation involved placing 5.0 g of material in headspace vials, with volatile compound enrichment achieved through solid-phase microextraction (SPME) using a 1 cm DVB/CAR/PDMS (divinylbenzene/carboxen/polydimethylsiloxane) triple-phase extraction fiber. The analytical protocol consisted of sample equilibration at 60 °C for 10 min, followed by headspace extraction at the same temperature for 40 min. Upon completion of the extraction phase, thermal desorption of the fiber was conducted in the GC injection port at 250 °C for 5 min prior to chromatographic analysis.

Comprehensive GC×GC-TOF-MS analysis employed a first-dimension column of DB-WAX (30 m × 250 μm × 0.25 μm) with a temperature program initiating at 40 °C (3 min hold), followed by a linear gradient of 5 °C/min to 250 °C (5 min hold). High-purity helium served as the carrier gas at a constant flow rate of 1.0 mL/min under splitless injection conditions. The second-dimensional column utilized a DB-17MS column (2 m × 100 μm × 0.10 μm) with both column and thermal modulator temperatures maintained at +5 °C relative to the first-dimension column. The modulation period was set to 6 s. Mass spectrometric detection employed electron ionization (EI) at 70 eV with source and transfer line temperatures maintained at 250 °C and 270 °C, respectively. Data acquisition encompassed the *m*/*z* range of 33–500 at an acquisition rate of 50 spectra per second with detector voltage set to 1680 V.

Raw spectral data underwent comprehensive preprocessing using R software (version 3.3.2), encompassing peak extraction, chromatographic alignment, noise reduction, and data normalization to generate a structured data matrix suitable for multivariate statistical analysis [36]. The preprocessed dataset was subsequently analyzed using the ropls package within the R environment, employing multiple complementary statistical approaches: principal component analysis (PCA), partial least squares discriminant analysis (PLS-DA), and orthogonal partial least squares discriminant analysis (OPLS-DA). Group-specific metabolite profiles were differentiated through OPLS-DA score plot visualization. Significantly altered metabolites were identified by integrating variable importance in VIP (Variable importance in project) > 1 with univariate statistical testing (Student’s *t*-test or one-way ANOVA, *p*-value (False Discovery Rate, FDR) < 0.05).

Volatile compound identification was accomplished through spectral matching against the National Institute of Standards and Technology (NIST) mass spectrometry database and the Wiley Registry metabolomics database [37]. To assess the sensory significance of individual aroma compounds, relative odor activity values (ROAVs) were calculated for each identified volatile organic compound. Compounds exhibiting ROAV ≥ 1 were classified as significant contributors to the overall aromatic profile and identified as key flavor-active substances. The ROAV was determined using the formula:

(1) $$ROAV=100\times \frac{C_{i}}{T_{i}}\times \frac{T_{max}}{C_{max}}$$

where *C_i_* represents the peak area of the target volatile compound, and *C_max_* represents that of the compound with the highest odor activity value; *T_i_* is the odor threshold in air, and *T_max_* is the largest odor threshold in air [38].

### 2.3. Metabolomics Analysis in M. laevigata

Sample preparation commenced with freeze-drying using a Scientz-100F system (Scientz, Ningbo, China), followed by mechanical homogenization to uniform powder using an MM 400 ball mill (Retsch, Shanghai, China) operating at 30 Hz. Precisely measured aliquots of 50 mg powder were subjected to metabolite extraction using 1200 μL of 70% aqueous methanol solution pre-chilled to −20 °C. The extraction protocol involved six cycles of vortex agitation (30 s at 30 min intervals), followed by centrifugation under refrigerated conditions. The resulting supernatant was clarified through 0.22 μm membrane filtration, and the clarified extract was retained for analysis.

Metabolite analysis was conducted using an integrated UPLC-ESI-MS/MS (UPLC-MS/MS, Thermo Fisher Scientific, Carlsbad, CA, USA). Chromatographic separation was achieved using an ACQUITY UPLC^®^ HSS T3 column (2.1 × 100 mm, 1.8 μm particle size; Waters, Milford, MA, USA). The analytical method operated in dual ionization modes with distinct mobile phase compositions. For positive ionization, the mobile phase consisted of 0.1% formic acid in acetonitrile (B2) and 0.1% aqueous formic acid (A2). For negative ionization, acetonitrile (B3) and 5 mM aqueous ammonium formate (A3) comprised the mobile phase system. The gradient elution program followed the established methodology of Zelena et al. [39]. Mass spectrometric detection employed electrospray ionization (ESI) with data acquisition conducted in both positive and negative ion modes. Source parameters included spray voltages of +3.50 kV and −2.50 kV for positive and negative modes, respectively, sheath gas flow at 40 arbitrary units, auxiliary gas at 10 arbitrary units, and capillary temperature maintained at 325 °C. Full-scan MS1 acquisition operated at 60,000 resolution across the *m*/*z* range of 100–1000. Tandem mass spectrometry employed higher-energy collisional dissociation (HCD) with 30% normalized collision energy and MS2 resolution of 15,000.

Raw spectral data underwent format conversion to mzXML using Proteowizard software (version 3.0.8789) [40]. Peak detection, filtering, and chromatographic alignment were performed using the R XCMS package with optimized parameters: bandwidth = 2, mass tolerance = 15 ppm, peak width range = 5–30 s, mass window = 0.015, mass difference threshold = 0.01, employing the centWave algorithm [41]. Metabolite identification was accomplished through spectral matching against comprehensive databases including the Human Metabolome Database (HMDB) [42], MassBank [43], LipidMaps [44], mzCloud [45], and the Kyoto Encyclopedia of Genes and Genomes (KEGG) [46], with mass accuracy tolerances maintained within 30 ppm. Quality assurance protocols involved the systematic exclusion of metabolites exhibiting relative standard deviation values exceeding 30% across quality control samples.

Multivariate statistical analysis was conducted using the R package ropls_1.4.0.0 [36], implementing principal component analysis (PCA), partial least squares discriminant analysis (PLS-DA), and orthogonal partial least squares discriminant analysis (OPLS-DA). Data preprocessing included appropriate scaling transformations, with model performance evaluated through variance metrics including R^2^X, R^2^Y, and Q^2^ parameters. Model validation employed permutation testing protocols to ensure statistical robustness. Significantly altered metabolites were identified by integrating variable importance in VIP > 1, Student’s t-test results with significance thresholds of *p*-value < 0.05, and fold change analysis. Identified differential metabolites underwent comprehensive pathway enrichment and network topology analysis using the MetaboAnalyst platform [47], with metabolic pathway visualization generated through KEGG Mapper integration.

### 2.4. RNA-Seq Analysis in M. laevigata

Total RNA extraction was performed using the Plant RNA Extraction Kit (Ambion, Thermo Fisher Scientific, Carlsbad, CA, USA) following standard protocols. RNA sequencing libraries were prepared using the NEBNext^®^ Ultra™ RNA Library Prep Kit (Illumina^®^, New England Biolabs, Ipswich, MA, USA) according to manufacturer specifications. cDNA library sequencing was conducted by Suzhou Panomik Biomedical Technology Co., Ltd. (Suzhou, Jiangsu, China) using the Illumina HiSeq platform. Raw sequencing data underwent comprehensive quality control filtering to eliminate adapter sequences, poly-N-containing reads, and low-quality sequences. The quality-filtered reads were subsequently aligned against the mulberry reference genome (https://ftp.ncbi.nlm.nih.gov/genomes/all/GCF/000/414/095/GCF_000414095.1_ASM41409v2/, accessed on 26 May 2023) using the HISAT2-Align plugin within the TBtools software suite [48]. Gene expression quantification was conducted using the fragments per kilobase of transcript per million mapped reads (FPKM) methodology [49]. Differential expression analysis was performed using the DESeq2 package within the R statistical environment to identify differentially expressed genes [50]. Genes meeting the following criteria were classified as significantly differentially expressed: adjusted *p*-value < 0.05 following multiple testing correction and absolute |log_2_ (foldchange)| ≥1. Functional annotation of differentially expressed genes was conducted through Gene Ontology enrichment analysis and Kyoto Encyclopedia of Genes and Genomes pathway analysis using the TBtools software platform (v2.307) [48].

Based on the expression profiles of key differential genes within critical metabolic pathways, selected genes of particular interest were subjected to RT-qPCR validation across four experimental conditions (HF, MT, YMR, G4). The experimental design incorporated three biological replicates for each condition, with four technical replicates performed for each biological replicate to ensure robust statistical analysis and data reliability. Total RNA was extracted from freshly harvested mature mulberry fruits using the RNA simple Total RNA Kit (TIANGEN, Beijing, China). cDNA synthesis was subsequently performed using the TransScript-Uni One-Step gDNA Removal and cDNA Synthesis SuperMix kit (TransGen Biotech, Beijing, China) following manufacturer protocols. Quantitative real-time PCR (qRT-PCR) analysis was conducted using the Applied Biosystems 7500 Real-Time PCR System (Thermo Fisher, Carlsbad, CA, USA) with SYBR Premix Ex Taq reagent (TaKaRa, Tokyo, Japan) in a two-step amplification protocol. Thermal cycling parameters comprised initial denaturation at 95 °C for 7 min, followed by 40 cycles of 95 °C for 15 s, 60 °C for 30 s, and 72 °C for 30 s. Gene expression was normalized using ACTB as the internal reference, with primer sequences provided in Table S1. Relative expression levels were calculated using the 2^−ΔΔCt^ method [51].

### 2.5. Statistical Analysis

Statistical analyses were conducted using Microsoft Excel 2019. Each analysis incorporated a minimum of three independent biological replicates. Graphical representations were generated using GraphPad Prism 8.3.0 and TBtools v2.307, with final formatting completed in Adobe Illustrator 2020.

## 3. Result

### 3.1. Volatile Compounds in Ripened Fruits

Comprehensive GC × GC-TOF MS analysis was employed to characterize volatile metabolites across four *M. laevigata*. The resulting two-dimensional (Figure S1) and three-dimensional total ion chromatograms (Figure S2) demonstrated robust peak resolution and signal intensity for each sample, confirming the presence of diverse volatile compound profiles in all analyzed materials. The robust chromatographic peak profiles observed across all samples demonstrated the presence of abundant volatile metabolites in each *M. laevigata*. The analytical results revealed 312, 344, 393, and 326 volatile compounds in the respective *M. laevigata*. The most abundant chemical classes comprised esters, lipids and lipid-like molecules, heterocyclic compounds, ketones, hydrocarbons, aldehydes, alcohols, and benzenoids. Notable inter-accession variations were observed in the abundance of esters, lipids and lipid-like molecules, hydrocarbons, and alcohols, with ‘YMR’ demonstrating significantly higher concentrations of these compound classes (Table 1).

### 3.2. Characterization of Key Volatile Compounds and Aroma Profiles in M. laevigata

Orthogonal partial least squares discriminant analysis successfully discriminated among the four accessions, based on latent variable scores, achieving statistically significant separation (*p* < 0.05, R^2^X = 0.642, R^2^Y = 0.994, Q^2^ = 0.899). Model validation through permutation testing was conducted to assess predictive capacity and mitigate potential overfitting, thereby ensuring robust statistical performance across independent datasets. The permutation validation yielded y-intercepts of R^2^ = 0.97 and Q^2^ = −0.06, confirming the statistical validity and predictive reliability of the OPLS-DA classification model (Figure S3).

**Figure 2:**
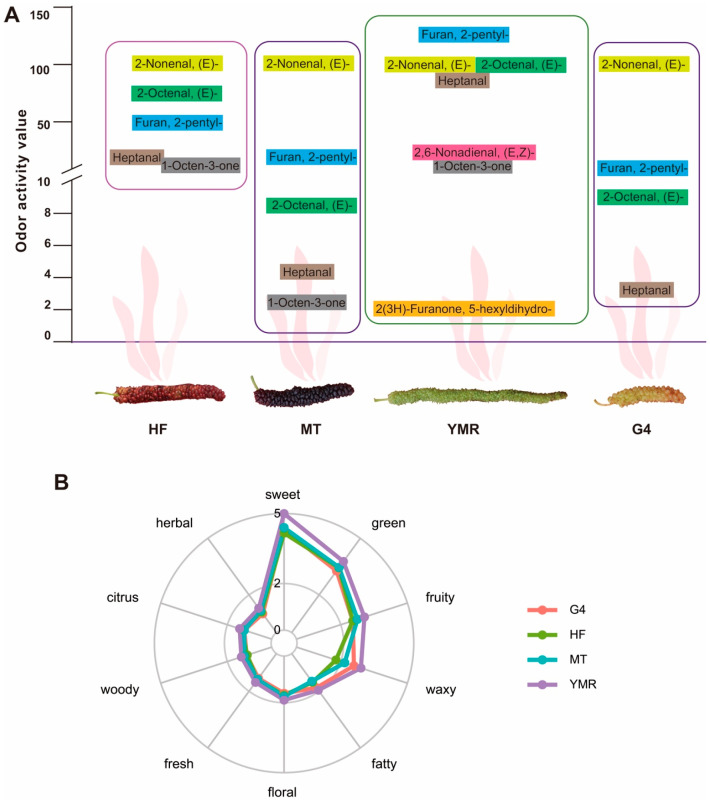
Key volatile compounds and their aroma profiles in four *M. laevigata*: (**A**) Contribution of volatiles based on odor activity values (ROAV ≥ 1); compounds within each mulberry are ranked in descending order of ROAV. (**B**) Sensory aroma radar plots illustrating dominant flavor attributes of the four *M. laevigata*; outer ring labels denote sensory descriptors derived from aroma evaluation.

Odor activity values (ROAVs) provide quantitative assessment of individual aroma compounds’ contributions to the overall sensory profile of fruit samples. Analysis revealed 5, 5, 7, and 4 aroma-active volatile compounds (ROAV ≥ 1) in ‘HF’, ‘MT’, ‘YMR’, and ‘G4’, respectively (Figure 2A). These compounds were primarily classified within the chemical classes of aldehydes, heterocyclic compounds, and ketones. These volatile compounds contributed distinctive sensory descriptors including fruity, green, vegetable, fatty, sweet, buttery, strong, and waxy notes to the four *M. laevigata* profiles (Table S2). (*E*)-2-Nonenal, (*E*)-2-octenal, and 2-pentylfuran consistently ranked among the three highest contributors across all four *M. laevigata*, suggesting their role as principal aroma-active compounds in this species (Figure 2A). Notably, (*E*, *Z*)-2,6-nonadienal and 2(3*H*)-furanone, 5-hexyldihydro exhibited odor activity values (ROAV ≥ 1) in ‘YMR’. Comprehensive sensory annotation of all detected volatile compounds across the four *M. laevigata* resulted in the identification of ten primary flavor categories: sweet, green, fruity, waxy, fatty, floral, fresh, woody, citrus, and herbal (Figure 2B). While the four *M. laevigata* demonstrated comparable aroma profiles, ‘YMR’ exhibited superior overall aromatic intensity compared to ‘HF’, ‘MT’, and ‘G4’, indicating enhanced flavor complexity in this accession.

### 3.3. Correlation Analysis Between Key Volatile Compounds and Primary and Secondary Metabolites in M. laevigata

**Figure 3:**
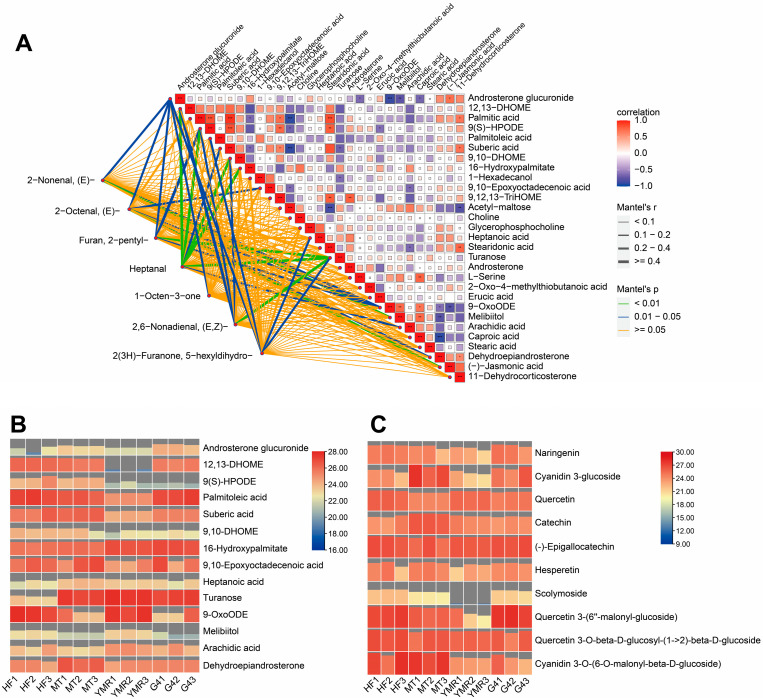
Integrated correlation analysis of volatile compounds and metabolites, and hierarchical clustering heatmap of differentially accumulated metabolites associated with key volatiles: (**A**) Correlation network between volatiles and metabolites. Metabolites were selected based on |log_2_FC| ≥ 1 and VIP > 1. (**B**) Heatmap of relative abundances for sugar- and fatty acid pathway metabolites significantly correlated with volatile compounds. (**C**) Heatmap of relative abundances for flavonoid pathway metabolites significantly correlated with volatile compounds. Asterisks denote statistical significance levels (* *p* < 0.05; ** *p* < 0.01;*** *p* < 0.001; Student’s *t*-test.

Analysis revealed that all seven key volatile compounds originated from fatty acid metabolism pathways. Furthermore, comparative analysis demonstrated that differential metabolites in ‘YMR’ relative to ‘HF’, ‘MT’, and ‘G4’ showed consistent enrichment in fatty acid metabolism pathways (Figure S4). Consequently, Mantel test analysis was performed to examine correlations between key volatile compounds and fatty acid pathway-related differential metabolites meeting significance criteria (|log_2_FC| > 1, VIP > 1) (Figure 3A). The analysis revealed significant correlations between sugar metabolism-related metabolites and multiple volatile compounds. Androsterone glucuronide demonstrated significant associations with (*E*)-2-nonenal, (*E*)-2-octenal, 2-pentylfuran, 1-octen-3-one, and 2(3*H*)-furanone, 5-hexyldihydro. Similarly, turanose exhibited significant correlations with 2-pentylfuran, (*E*, *Z*)-2,6-nonadienal, and heptanal. Among fatty acid metabolism-related metabolites, both 12, 13-DHOME and 9, 10-DHOME showed significant correlations with (*E*, *Z*)-2,6-nonadienal and heptanal. Additionally, 9-OxoODE demonstrated significant associations with (*E*)-2-nonenal, (*E*)-2-octenal, and 2-pentylfuran. The lipoxygenase pathway metabolite 9(S)-HPODE exhibited significant correlations with 2-pentylfuran, (*E*, *Z*)-2,6-nonadienal, and heptanal.

Heatmap analysis illustrated the relative abundance of metabolites demonstrating significant associations with flavor profiles (Figure 3B). Sugar metabolism-associated compounds (turanose and melibiitol) and fatty acid pathway metabolites (16-hydroxypalmitate, 9-OxoODE, and arachidic acid) exhibited moderately elevated concentrations in ‘YMR’, relative to the other accessions. In contrast, the key fatty acid metabolism intermediate 9(*S*)-HPODE demonstrated reduced abundance in ‘YMR’, compared to the other accessions. This pattern likely reflects the extensive oxidative conversion of precursor metabolites to flavor-active compounds in ‘YMR’.

Consequently, analysis of antioxidant-related flavonoid metabolites was conducted to further investigate this relationship (Figure 3C). The analysis revealed consistently reduced concentrations of key flavonoid compounds in ‘YMR’, including naringenin, cyanidin 3-glucoside, hesperetin, scolymoside, quercetin 3-(6″-malonyl-glucoside), and cyanidin 3-O-(6-O-malonyl-β-D-glucoside), compared to the other accessions. These findings indicate that reduced antioxidant capacity in ‘YMR’ corresponds with enhanced oxidative activity, facilitating increased conversion of precursor metabolites to flavor-related volatile compounds. This metabolic profile likely accounts for the superior flavor intensity observed in ‘YMR’.

### 3.4. Transcript Abundance of Genes Associated with Fatty Acid and Flavonoid Biosynthetic Pathways in M. laevigata

To elucidate the molecular mechanisms underlying flavor development in *M. laevigata* and establish a theoretical framework for breeding programs targeting enhanced fruit quality, comprehensive transcriptomic analysis was conducted. RNA sequencing analysis was conducted across the four *M. laevigata*, resulting in the construction of 12 cDNA libraries. Quality assessment demonstrated robust sequencing performance, with Q20 scores exceeding 97.83% and Q30 scores surpassing 93.68%. These quality metrics confirm that the sequence datasets met established standards for comprehensive transcriptomic investigation. Principal component analysis demonstrated excellent reproducibility within biological replicates and clear differentiation between accessions, confirming the statistical robustness of the transcriptomic data (Figure S5A). Differential expression analysis using stringent criteria (|log_2_FC| ≥ 1, FDR < 0.05) identified 2971 differentially expressed genes in ‘YMR’ vs. ‘HF’ (1295 upregulated, 1676 downregulated), 2334 genes in ‘YMR’ vs. ‘MT’ (1166 upregulated, 1168 downregulated), and 3406 genes in ‘YMR’ vs. ‘G4’ (1487 upregulated, 1919 downregulated) (Figure S5B). Pathway enrichment analysis revealed significant overrepresentation of differentially expressed genes in fundamental metabolic processes, including energy metabolism, carbohydrate metabolism, and secondary metabolite biosynthesis. Notably, all pairwise comparisons between ‘YMR’ and the other *M. laevigata* consistently demonstrated enrichment in pathways directly relevant to flavor development. These included fatty acid elongation, fatty acid biosynthesis, α-linolenic acid metabolism, and flavonoid biosynthesis (Figure S5C–E). These metabolic processes likely contribute to the distinctive aromatic profile observed in ‘YMR’.

**Figure 4:**
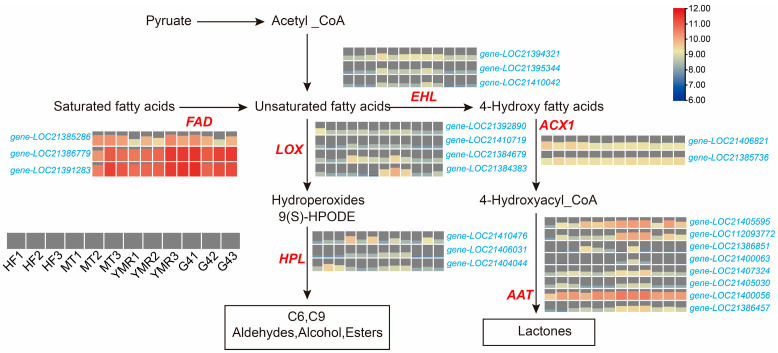
Heatmap illustrating transcript abundance of key genes associated with the fatty acid metabolic pathway in four *M. laevigata*. Genes include lipoxygenase (*LOX*), hydroperoxide lyase (*HPL*), fatty acid desaturase (*FAD*), epoxide hydrolase (*EHL*), acyl-CoA oxidase 1 (*ACX1*), and alcohol acyltransferase (*AAT*).

Gene expression analysis within fatty acid metabolism pathways was conducted to generate comprehensive pathway expression maps (Figure 4). Lipoxygenase expression patterns demonstrated clear accession-specific profiles. *LOX3* (*gene-LOC21384383*) exhibited pronounced upregulation in ‘YMR’, while two distinct *LOX5* isoforms showed differential expression: *gene-LOC21384679* displayed elevated expression in both ‘YMR’ and ‘MT’, whereas *gene-LOC21392890* was expressed across multiple accessions, including ‘YMR’, ‘HF’, and ‘MT’. Hydroperoxide lyase (*HPL*) expression was detected across all four *M. laevigata*, potentially explaining the elevated odor activity values observed for (*E*)-2-nonenal, (*E*)-2-octenal, and 2-pentylfuran in *M. laevigata* (Figure 2A).

Genes associated with lactone biosynthesis demonstrated consistently elevated transcriptional activity in ‘YMR’. Specific enzyme families exhibited distinct expression patterns across *M. laevigata* fatty acid desaturase (*FAD*) genes (*gene-LOC21386779*, *gene-LOC21391283*) showed pronounced upregulation in ‘YMR’. Enoyl-CoA hydratase (*EHL*) genes (*gene-LOC21394321*, *gene-LOC21395344*, *gene-LOC21410042*) demonstrated enhanced expression in both ‘YMR’ and ‘MT’. Acyl-CoA oxidase (*ACX1*) (*gene-LOC21385736*) exhibited elevated expression in ‘YMR’ and ‘G4’. Alcohol acyltransferase (*AAT*) genes (*gene-LOC21405595*, *gene-LOC112093772*, *gene-LOC21407324*, *gene-LOC21400056*, *gene-LOC21386457*) displayed preferential expression in ‘YMR’. These findings reveal a notable paradox whereby ‘YMR’ exhibits reduced metabolite accumulation within fatty acid metabolism pathways compared to other *M. laevigata* (Figure 3B) yet demonstrates consistently elevated transcriptional activity of associated enzyme genes (Figure 4). This pattern indicates enhanced metabolic flux and oxidative conversion efficiency in ‘YMR’, facilitating increased production of volatile flavor compounds.

**Figure 5:**
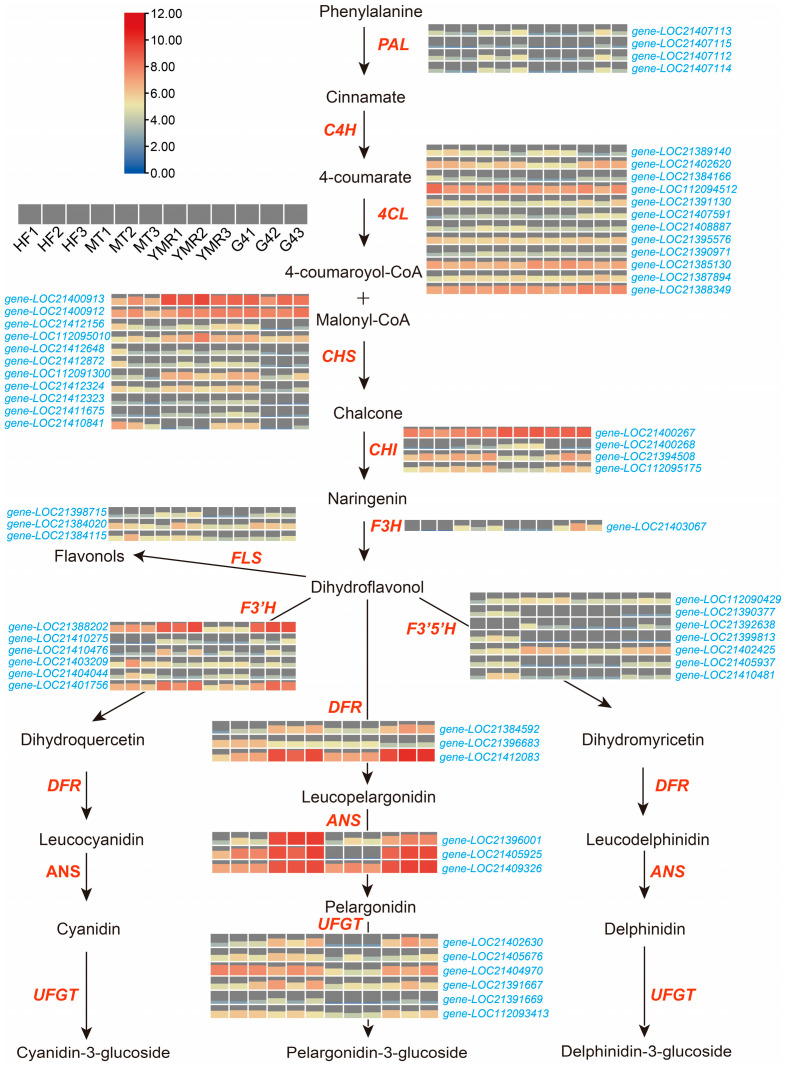
Heatmap depicting transcript levels of key biosynthetic genes in the phenylpropanoid pathway across four *M. laevigata*. Genes include: phenylalanine ammonia-lyase (*PAL*), cinnamate 4-hydroxylase (*C4H*), 4-coumarate:CoA ligase (*4CL*), chalcone synthase (*CHS*), chalcone isomerase (*CHI*), flavanone 3-hydroxylase (*F3H*), flavonoid 3′-hydroxylase (*F3′H*), flavonoid 3′,5′-hydroxylase (*F3′5′H*), dihydroflavonol 4-reductase (*DFR*), anthocyanidin synthase (*ANS*), and UDP-glucose flavonoid 3-O-glucosyltransferase (*UFGT*).

Comprehensive expression analysis of phenylpropanoid biosynthesis pathway genes was performed across the four *M. laevigata* (Figure 5). Key upstream enzymes demonstrated distinct expression profiles across accessions. Phenylalanine ammonia-lyase (*PAL*) genes (*gene-LOC21407113*, *gene-LOC21407112*, *gene-LOC21407114*) exhibited elevated expression in ‘HF’, ‘MT’, and ‘G4’. 4-Coumarate-CoA ligase (*4CL*) genes (*gene-LOC21402620*, *gene-LOC112094512*, *gene-LOC21395576*, *gene-LOC21385130*, *gene-LOC21387894*, *gene-LOC21388349*) showed ubiquitous expression across all four *M. laevigata*. Downstream enzymes in the flavonoid biosynthesis pathway exhibited variable expression patterns. Chalcone synthase (*CHS*) genes (*gene-LOC21400913*, *gene-LOC21400912*, *gene-LOC112095010*, *gene-LOC112091300*) demonstrated preferential expression in ‘MT’ and ‘YMR’. Chalcone isomerase (*CHI*) genes (*gene-LOC21400267*, *gene-LOC21394508*, *gene-LOC112095175*) displayed elevated expression specifically in ‘HF’, ‘MT’, and ‘G4’. These upstream enzymatic activities establish the metabolic foundation for subsequent flavonoid biosynthesis pathways.

Analysis of downstream phenylpropanoid pathway components revealed distinct expression patterns among key regulatory enzymes. Flavanone 3-hydroxylase (*F3H*), (*gene-LOC21403067*) demonstrated elevated expression in ‘MT’ and ‘G4’ accessions, while flavonol synthase (*FLS*) genes (*gene-LOC21398715*, *gene-LOC21384020*, *gene-LOC21384115*) exhibited consistently reduced expression in ‘YMR’. Critical flavonoid biosynthesis enzymes demonstrated substantial expression differences across accessions. Flavonoid 3′-hydroxylase (*F3′H*) genes (*gene-LOC21388202*, *gene-LOC21410275*, *gene-LOC21410476*, *gene-LOC21403209*, *gene-LOC21401756*), flavonoid 3′,5′-hydroxylase (*F3′5′H*) genes (*gene-LOC112090429*, *gene-LOC21390377*, *gene-LOC21399813*, *gene-LOC21402425*, *gene-LOC21405937*, *gene-LOC21410481*), and UDP-glucose flavonoid 3-O-glucosyltransferase (*UFGT*) genes (*gene-LOC21402630*, *gene-LOC21405676*, *gene-LOC21404970*, *gene-LOC21391667*, *gene-LOC112093413*) exhibited markedly higher expression in ‘HF’, ‘MT’, and ‘G4’ relative to ‘YMR’. Additionally, dihydroflavonol 4-reductase (*DFR*) genes (*gene-LOC21384592*, *gene-LOC21412083*) and anthocyanidin synthase (*ANS*) genes (*gene-LOC21396001*, *gene-LOC21405925*, *gene-LOC21409326*) displayed preferential expression in ‘MT’ and ‘G4’ accessions. These expression profiles demonstrate that ‘HF’, ‘MT’, and ‘G4’ possess enhanced phenylpropanoid biosynthetic capacity compared to ‘YMR’, characterized by elevated enzyme gene expression and correspondingly greater flavonoid production potential. This genetic architecture aligns with the observed higher flavonoid metabolite concentrations in these three *M. laevigata* (Figure 3C).

### 3.5. qRT-PCR Validation of DEGs

**Figure 6:**
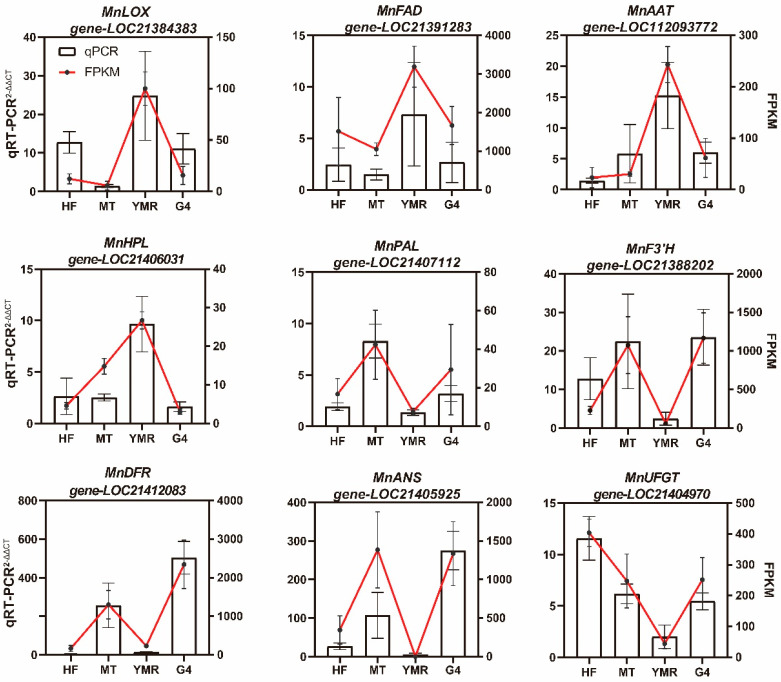
Transcriptional levels of candidate genes by qRT-PCR. The left *Y*-axis shows relative expression values calculated using the 2^−ΔΔCT^ method; the right *Y*-axis displays corresponding transcript abundance from RNA-Seq data as FPKM.

To further validate the accuracy of the transcriptomic data, we selected key biosynthetic genes associated with fatty acid metabolism (*MnLOX*, *MnHPL*, *MnFAD*, *MnAAT*) and phenylpropanoid metabolism (*MnPAL*, *MnF3′H*, *MnDFR*, *MnANS*, *MnUFGT*) in *Morus* L. for qRT-PCR analysis (Figure 6). Expression analysis revealed that *MnLOX* (*gene-LOC21384383*), which catalyzes unsaturated fatty acid oxidation to hydroperoxides, and *MnHPL (gene-LOC21406031)*, responsible for hydroperoxide conversion to volatile compounds, were both significantly upregulated in ‘YMR’ relative to ‘HF’, ‘MT’, and ‘G4’. Lactone biosynthesis genes *MnFAD (gene-LOC21391283)* and *MnAAT (gene-LOC112093772)* similarly exhibited elevated expression in ‘YMR’. Conversely, anthocyanin biosynthesis genes *MnPAL (gene-LOC21407112)*, *MnF3’H (gene-LOC21388202)*, *MnDFR (gene-LOC21412083)*, *MnANS (gene-LOC21405925)*, and *MnUFGT (gene-LOC21404970)* demonstrated significantly higher expression in ‘HF’, ‘MT’, and ‘G4’ compared to ‘YMR’. These findings demonstrate strong concordance between qRT-PCR and transcriptomic data, thereby validating the reliability of the transcriptomic analysis.

## 4. Discussion

Mulberry fruit flavor profiles are governed by the complex interplay of sugars, organic acids, and diverse volatile compounds including aldehydes, esters, alcohols, and terpenes [52]. This investigation conducted comprehensive volatile compound analysis across four *M. laevigata* (‘HF’, ‘MT’, ‘G4’, and ‘YMR’). The analysis identified 312, 344, 393, and 326 volatile compounds in the respective accessions, distributed across 15 distinct chemical categories. The most abundant compound classes included esters, lipids and lipid-like molecules, heterocyclic compounds, ketones, hydrocarbons, aldehydes, alcohols, and benzenoids (Table 1). These volatile constituents form the fundamental basis of mulberry fruit flavor architecture, demonstrating the remarkable chemical complexity and diversity underlying sensory perception in this species.

Aldehydes represent critical volatile compounds in fruit flavor development, contributing distinctive sensory characteristics including fresh, apple-like, and citrus peel notes [53]. These compounds contribute grassy notes that enhance perceived freshness in tomatoes [54], while C6 aldehydes serve as key determinants of kiwi fruit flavor profiles, imparting characteristic green and grassy sensory attributes to green-fleshed varieties [55]. In peach varieties, aldehydes constitute primary aroma-active compounds, with heptanal, (*E*, *E*)-2,6-nonadienal, and octanal contributing distinctive green and citrus flavor characteristics [56]. The present analysis identified four aldehydes with significant odor activity values (ROAV ≥ 1) in four *M. laevigata*: (*E*)-2-nonenal, (*E*)-2-octenal, heptanal, and (*E*, *Z*)-2,6-nonadienal (Figure 2A), demonstrating their substantial contribution to *M. laevigata* flavor profiles. (*E*)-2-Nonenal serves as a principal component in natural fragrance formulations, exhibiting a complex sensory profile encompassing fresh, fatty, and green aromatic notes [57]. This compound demonstrated consistently elevated odor activity values (ROAV = 100) across all four *M. laevigata* (Table S2), establishing its role as an essential contributor to the distinctive aromatic character of *M. laevigata* (*E*)-2-Octenal and heptanal, providing substantial aromatic contributions, typically associated with tea-like and grape-derived notes [58]. (*E*, *Z*)-2,6-Nonadienal exhibits a multifaceted sensory profile characterized by green, sweet, and melon-like attributes, representing a significant flavor-active compound across numerous fruit species [59].

Ketones arise primarily through lipid oxidation processes, contributing distinctive sensory characteristics including floral and mushroom-like notes [60]. Specific ketone compounds, including 1-penten-3-one, 2-nonanone, and 2-octanone, impart characteristic flavor profiles to mulberry fruits, notably vanilla, floral, and caramel sensory attributes [61]. 1-Octen-3-one has been identified as a critical aroma-active compound contributing significantly to tomato flavor complexity [62]. The present analysis demonstrated that 1-octen-3-one exhibited odor activity values (ROAV ≥ 1) in ‘HF’, ‘MT’, and ‘YMR’ (Figure 2A), establishing its meaningful contribution to the overall aromatic profile of *M. laevigata.*

2-Pentylfuran originates from linoleic acid degradation pathways and exhibits characteristic green and fatty aromatic properties [63]. This compound has been recognized as a critical aroma-active constituent in diverse food matrices, including stone fruits such as plums [35] and various mushroom species [64]. The present analysis demonstrated that 2-pentylfuran consistently ranked among the three highest contributors across all four *M. laevigata* (Figure 2A), establishing its significant role in defining the characteristic aromatic profile of *M. laevigata*.

2(3*H*)-furanone,5-hexyldihydro represents a significant lactone compound identified as a major volatile constituent in peach varieties, exhibiting a complex sensory profile characterized by peach, fresh, coconut, sweet, and buttery aromatic attributes. This compound finds extensive commercial applications across food manufacturing and fragrance industries [65]. The biosynthetic pathway of 2(3*H*)-furanone,5-hexyldihydro involves sequential enzymatic transformations mediated by fatty acid desaturase (*FAD*), enoyl-CoA hydratase (*EHL*), acyl-CoA oxidase (*ACX1*), and alcohol acyltransferase (*AAT*). This complex process encompasses dehydrogenation, epoxidation, hydration, hydroxylation, and β-oxidative chain shortening of saturated fatty acid precursors, culminating in lactone ring formation [66]. Previous research has identified 2(3*H*)-furanone,5-hexyldihydro as the predominant aromatic constituent in ‘Baichang’ mulberry cultivars, contributing distinctive creamy sensory characteristics [32]. The present analysis revealed that 2(3*H*)-furanone, 5-hexyldihydro exhibited odor activity values (ROAV ≥ 1) exclusively in the ‘YMR’ (Figure 2A). Correspondingly, the majority of genes governing lactone biosynthesis, including *FAD*, *EHL*, *ACX1*, and *AAT*, demonstrated elevated transcriptional activity specifically in ‘YMR’ (Figure 4). These findings suggest that 2(3*H*)-furanone, 5-hexyldihydroserves as a critical determinant of the distinctive aromatic profile characteristic of the ‘YMR’.

All aroma-active volatile compounds exhibiting ROAV ≥ 1 in *M. laevigata* derive from fatty acid catabolism and subsequent esterification processes. Fatty acid metabolism serves as a fundamental determinant in food flavor development, representing an essential biochemical process that cannot be substituted in aromatic compound generation [67,68]. The majority of flavor-active compound classes, including aldehydes, alcohols, esters, ketones, and lactones, arise through three distinct metabolic processes within fatty acid catabolism: α-oxidation, β-oxidation, and the lipoxygenase pathway [69]. The lipoxygenase pathway represents the central enzymatic mechanism governing the oxidative catabolism of unsaturated fatty acids, facilitating the biosynthesis of volatile flavor compounds [70]. This process involves the lipoxygenase-mediated conversion of unsaturated fatty acids to 9/13-(*S*)-hydroperoxyoctadecadienoic acid intermediates, which subsequently undergo stereospecific enzymatic cleavage by hydroperoxide lyase to yield diverse volatile metabolites [71]. Lipoxygenase genes exhibit widespread distribution across plant species, with varying gene copy numbers and functional specialization observed among different taxa [72]. Regarding volatile compound biosynthesis, peach fruit maturation is characterized by downregulation of *PpLOX2* and *PpLOX3* expression, coinciding with reduced accumulation of key aroma compounds including hexanal, (*E*)-2-hexenal, (*E*)-2-hexenol, and (*Z*)-3-hexenol [73]. Furthermore, in strawberry (*Fragaria* spp.), *FaLOX5* functions as a key regulatory enzyme governing C6 volatile compound biosynthesis [74]. The analysis demonstrates that predominant volatile compounds in four *M. laevigata*, including (*E*)-2-nonenal, (*E*, *Z*)-2,6-nonadienal, (*E*)-2-octenal, heptanal, 1-octen-3-one, and 2-pentylfuran, derive from unsaturated fatty acid oxidation pathways. Transcriptomic analysis revealed elevated expression of three *LOX* genes (*gene-LOC21384383*, *gene-LOC21384679*, *gene-LOC21392890*) in ‘YMR’. Additionally, *FAD*, the primary gene encoding fatty acid desaturase for unsaturated fatty acid biosynthesis, exhibited high expression in ‘YMR’, along with lactone biosynthesis pathway genes *EHL*, *ACX1*, and *AAT*. Notably, despite elevated lipoxygenase pathway gene expression in the superior-flavored ‘YMR’ accession (Figure 4), the relative abundance of the key metabolic intermediate 9(*S*)-HPODE was reduced (Figure 3B). This apparent paradox can be attributed to enhanced metabolic flux in ‘YMR’. Elevated *FAD* expression increases unsaturated fatty acid precursor availability, while heightened *LOX* activity accelerates precursor oxidation, resulting in efficient conversion to volatile aldehydes and heterocyclic compounds. Concurrently, *EHL*, *ACX1*, and *AAT* activity facilitates lactone volatile formation.

Lipid oxidation represents a fundamental biochemical process governing plant flavor development, proceeding through distinct mechanistic pathways including auto-oxidation, photo-oxidation, and enzyme-mediated oxidation [75]. Within this enzymatic framework, lipoxygenase serves as the primary catalyst facilitating controlled oxidation processes, while lipases function through hydrolytic mechanisms to liberate free fatty acids, thereby establishing the substrate pool essential for downstream oxidative transformations. Through lipoxygenase-mediated catalysis, polyunsaturated fatty acids including linoleic acid and α-linolenic acid undergo oxidative transformation to form conjugated hydroperoxide intermediates, which subsequently experience enzymatic cleavage to yield short-chain volatile carbonyl compounds. Representative products include (*E*, *Z*)-2, 6-nonadienal, which imparts characteristic cucumber-like sensory attributes, and (*E*)-2-hexenal, associated with tomato-derived aromatic notes. These aldehyde compounds undergo subsequent reduction to their corresponding alcohol derivatives, (*E*, *Z*)-2,6-nonadienol and (*E*)-2-hexenol, which demonstrate reduced olfactory thresholds and enhanced aromatic intensity [76]. Additionally, non-enzymatic auto-oxidation processes contribute significantly to flavor compound development. Linoleic acid undergoes spontaneous oxidative reactions in aerobic conditions, generating 9/13-hydroperoxide intermediates that subsequently decompose to yield characteristic volatile products including hexanal, (*E*)-2-octenal, and (*E*, *E*)-2,4-decadienal [77]. Photo-oxidation proceeds at significantly accelerated rates compared to auto-oxidation processes and occurs through initiation by ultraviolet radiation and photosensitizing agents. The resulting alkyl radicals generated from fatty-acid–sensitizer interactions undergo subsequent reactions with molecular oxygen to form peroxy radicals. These intermediates propagate oxidative chain reactions through free radical mechanisms, ultimately yielding diverse hydroperoxide species that decompose to produce an array of volatile compounds [78]. The findings indicate that enhanced lipoxygenase gene activity drives increased oxidative processing of precursor compounds in ‘YMR’. However, lipid oxidation represents a multifaceted biochemical process where non-enzymatic oxidation pathways may contribute substantially to the accelerated oxidative transformations and abundant flavor compound production observed in ‘YMR’.

Antioxidant abundance and activity serve as critical regulatory factors governing both the extent and progression of oxidative processes, establishing essential equilibrium between flavor development and product quality preservation [79]. Flavonoids represent a diverse class of polyphenolic secondary metabolites ubiquitous throughout plant tissues, encompassing distinct structural subclasses including flavones, flavonols, flavanones, isoflavones, anthocyanins, and proanthocyanidins. The abundance of phenolic hydroxyl groups within flavonoid molecular architectures confers substantial antioxidant properties, enabling these compounds to function as effective free radical scavengers, lipid peroxidation inhibitors, and transition metal chelating agents [80,81]. Metabolomic analysis revealed significantly elevated flavonoid-related metabolite levels in ‘HF’, ‘MT’, and ‘G4’ accessions compared to ‘YMR’ (Figure 3C). Correspondingly, phenylpropanoid pathway genes demonstrated substantial upregulation in these three *M. laevigata*, particularly the anthocyanin biosynthesis genes *MnPAL*, *MnF3′H*, *MnDFR*, *MnANS*, and *MnUFGT* (Figure 5 and Figure 6). These findings indicate enhanced flavonoid biosynthetic capacity in ‘HF’, ‘MT’, and ‘G4’ accessions. The pronounced pigmentation differences observed among mulberries (Figure 1) indicate that anthocyanin accumulation serves as the primary driver of phenotypic color variation. Given that anthocyanins represent specialized flavonoid derivatives, their elevated concentrations in ‘HF’, ‘MT’, and ‘G4’ provide compelling evidence for enhanced antioxidant capacity within these accessions. Conversely, ‘YMR’ exhibits reduced flavonoid metabolite concentrations and diminished phenylpropanoid pathway gene expression, resulting in compromised antioxidant defense mechanisms. This biochemical profile renders the accession more vulnerable to oxidative stress, potentially accelerating the degradation of lipids, pigments, and flavor compounds through oxidative processes.

## 5. Conclusions

The distinctive *M. laevigata* flavor profile comprises several key volatile compounds: aldehydes including (*E*)-2-nonenal, (*E*)-2-octenal, heptanal, and (*E*, *Z*)-2, 6-nonadienal; the ketone 1-octen-3-one; the heterocyclic compound 2-pentylfuran; and the lactone 2(3*H*)-furanone, 5-hexyldihydro. These volatile compounds originate from fatty acid oxidation and cyclization reactions. Flavonoid metabolites possessing antioxidant properties may suppress oxidative processes, consequently modulating volatile compound composition. The ‘YMR’ accession exhibits reduced flavonoid and anthocyanin levels, resulting in diminished antioxidant capacity. This biochemical profile facilitates enhanced fatty acid oxidation, yielding superior volatile compound production and establishing ‘YMR’ as valuable germplasm for flavor enhancement breeding programs. These findings elucidate the metabolic balance governing flavor compound development and provide valuable guidance for mulberry breeding strategies aimed at enhancing fruit quality.

## Figures and Tables

**Table 1:** Distribution of volatile compounds across chemical classes in the four *M. laevigata*.

Classification	HF	MT	YMR	G4
Esters	30	42	50	36
Lipids and lipid-like molecules	38	40	45	31
Heterocyclic_Compounds	30	32	32	42
Organic oxygen compounds	11	10	9	10
Organohalogen compounds	5	8	7	6
Organosulfur compounds	1	1	0	1
Organonitrogen compounds	3	7	3	2
Ketones	24	30	29	27
Hydrocarbons	32	32	52	38
Carboxylic_Acids	6	7	10	3
Aldehydes	31	27	33	31
Ethers	8	4	7	6
Alcohols	26	20	33	23
Benzenoids	45	55	49	45
Others	22	29	34	25
Total	312	344	393	326

## Data Availability

The original contributions presented in this study are included in the article/Supplementary Material. Further inquiries can be directed to the corresponding author.

## Supplementary Materials

The following supporting information can be downloaded at: https://www.mdpi.com/article/10.3390/foods14193309/s1. Table S1. Primers used for real-time quantitative PCR. Table S2. The odor activity values and odor character of major volatile compounds in four *M. laevigata*. Figure S1. Two-dimensional total ion current chromatogram derived from GC–MS analysis of volatile and semi-volatile metabolites in four *M. laevigata.* Figure S2. Three-dimensional total ion current (TIC) chromatograms derived from GC–MS analysis of volatile and semi-volatile metabolites in four *M. laevigata*. Figure S3. Orthogonal partial least squares discriminant analysis (OPLS-DA) of metabolomic profiles in four *M. laevigata*. Figure S4. KEGG pathway enrichment analysis of differentially expressed metabolites (DEMs) across four *M. laevigata*. Figure S5. Comprehensive transcriptomic analysis and differential gene expression profiling across four *M. laevigata*.
