# Supplementary material for: Integrated Multi-Omics Approaches Provide Novel Insights into the Mechanisms Underlying Signature Flavor Development in Mulberry Fruits
Source: Foods. 2025 Sep 24;14(19):3309. doi: 10.3390/foods14193309 (PMC12523777; doi:10.3390/foods14193309)
Supplement: Supplementary file 1 [file foods-14-03309-s001.zip › foods-3850653-supplementary.pdf]

Table S1. Primers used for real-time quantitative PCR.

Gene ID	Gene name	NCBI ID	Forward primer (5'-3')	Reverse primer (5'-3')
gene-LOC21384383	LOX	XM_024169684.1	TGGATTGAAAGGGATGCTTGG	AGCTCCTGGGACTCCTATTTC
gene-LOC21391283	FAD	XM_010107154.2	TGACAGCCTCAAGCGAGTC	GAATGAGTGTACGACAGAGCG
gene-LOC112093772	AAT	XM_024172897.1	AGTGTACTACTACCCGTTTGCC	TCCGAGTTGATCGAACCCG
gene-LOC21406031	HPL	XM_010106009.2	GTGGCAACACCTACACTTCAC	GGTTCCCAATCTGGTGGAGG
gene-LOC21407112	PAL	XM_024172966.1	TCACCGACCACTTGACGC	GCAGCCTTCATGTAAGAGCTG
gene-LOC21388202	F3'H	XM_010107524.2	GTTGCTACGGAAGATCACGTCC	CGAGCCTCTGTTTGCTAGGG
gene-LOC21412083	DFR	XM_024174542.1	AGCAACATGGGATCGGTGAG	GTGGCTCGGACCGTGTAAC
gene-LOC21405925	ANS	XM_010109764.1	GAGGTGATCCCGCAAGAGTAC	GCTCTTCAAGTCAATGGTCGG
gene-LOC21404970	UFGT	XM_024166225.1	CCATGTCAACCCCATGATGC	TCCTCTGGACCGGATCAGAC
	ACTB		GAGGGCCGTGTTCCCAGCATCGTC	TCTTTTTGATTGAGCCTCATCCCCT

Note: The primers used were designed using the AlleleID 6. F, forward primer; R, reverse primer.

Table S2. The odor activity values and Odor Character of major volatile compounds in four *M. laevigata*.

Volatile compounds	Class	Odor Character	HF	MT	YMR	G4
2-Nonenal,(E)-	Aldehydes	Fatty, Cucumber	100	100	100	100
2-Octenal, (E)-	Aldehydes	Green,Fatty,Green,Fresh, Waxy	74.65	8.49	100.45	9.01
Furan,2 pentyl-	Heterocyclic_ Compounds	Vegetable,Fruity, Green, Butter	49.45	22.16	127.36	11.98
Heptanal	Aldehydes	Green,Citrus,Fatty	15.74	4.20	86.15	3.24
1-Octen-3-one	Ketones	Fatty,Green,Fresh,Banana , Waxy	14.29	2.46	13.24	0.63
2,6-Nonadienal, (E,Z)-	Aldehydes	Violet, Wax, Green, Nutty, Fatty	0.66	0.81	22.25	0.48
2(3H)-Furanone, 5-hexyldihydro-	Heterocyclic_ Compounds	Fruity, Peach, Oily, Fresh, Strong, Coconut, Sweet, Fatty, Butter, Waxy	0.16	0.14	2.20	0.29

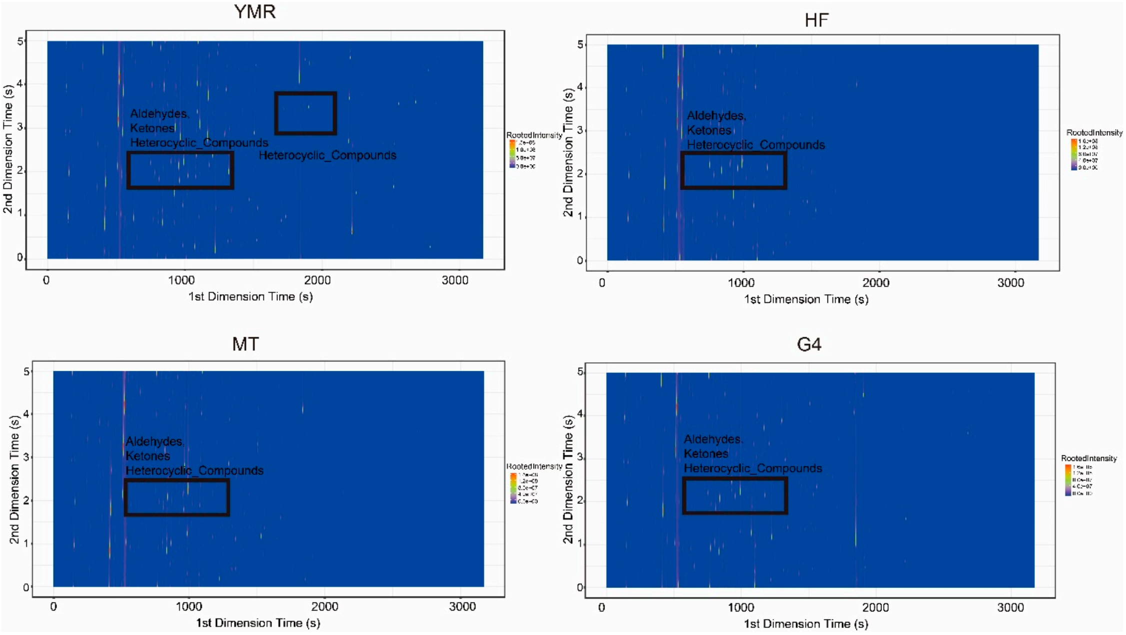

Figure S1. Two-dimensional total ion current chromatogram derived from GC–MS analysis of volatile and semi-volatile metabolites in four *M. laevigata*.

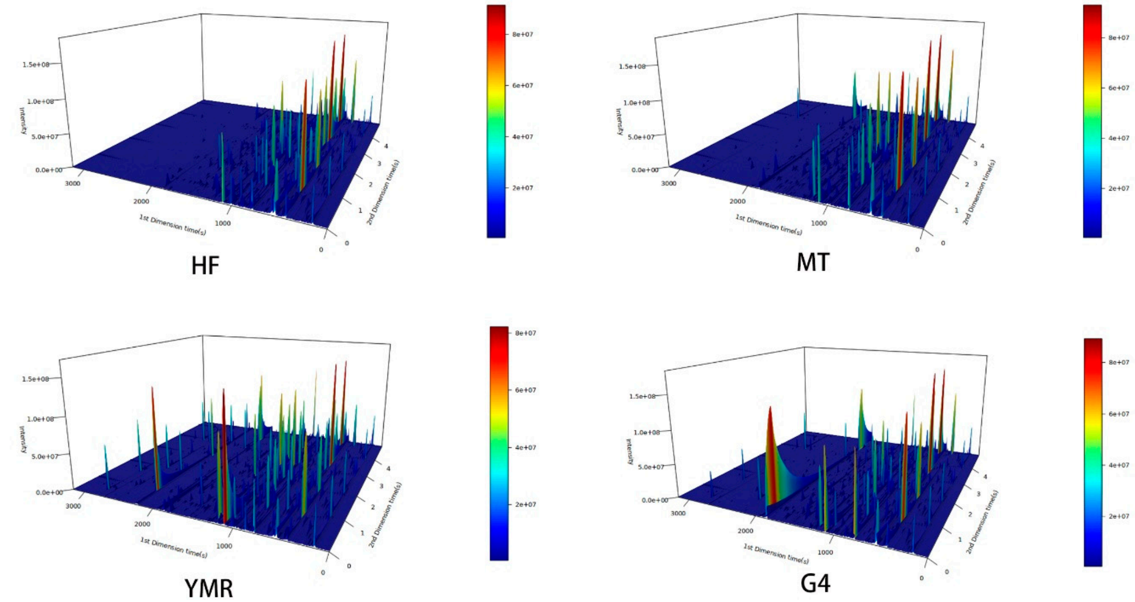

Figure S2. Three-dimensional total ion current (TIC) chromatograms derived from GC–MS analysis of volatile and semi-volatile metabolites in four *M. laevigata*.

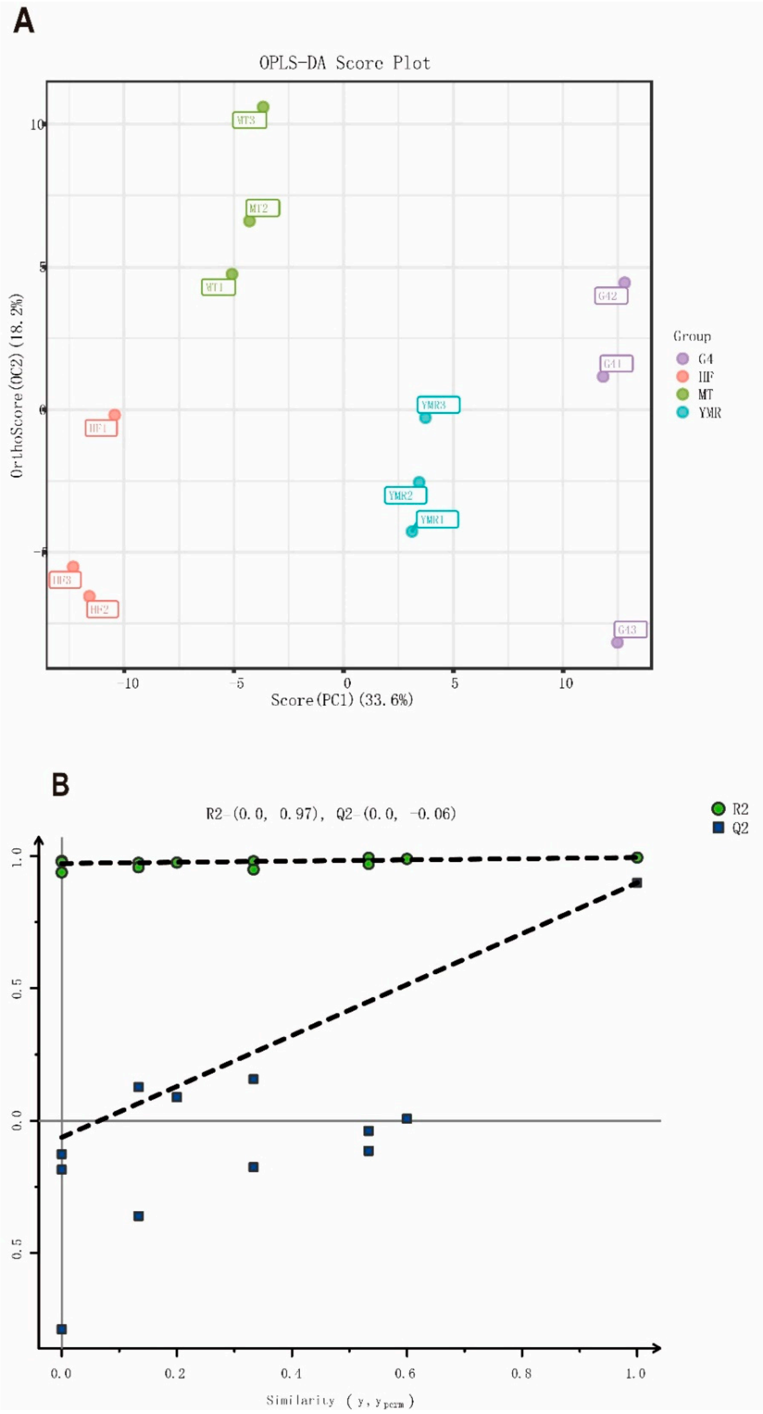

Figure S3. Orthogonal partial least squares discriminant analysis (OPLS-DA) of metabolomic profiles in four *M. laevigata.* (A) Scores plot showing clear separation between sample groups, with model parameters $R^2X = 0.642$, $R^2Y = 0.994$, and $Q^2 = 0.899$; (B) Permutation test results, with $R^2 = (0.0 , 0.97)$, $Q^2 =(0.0 , –0.060)$.

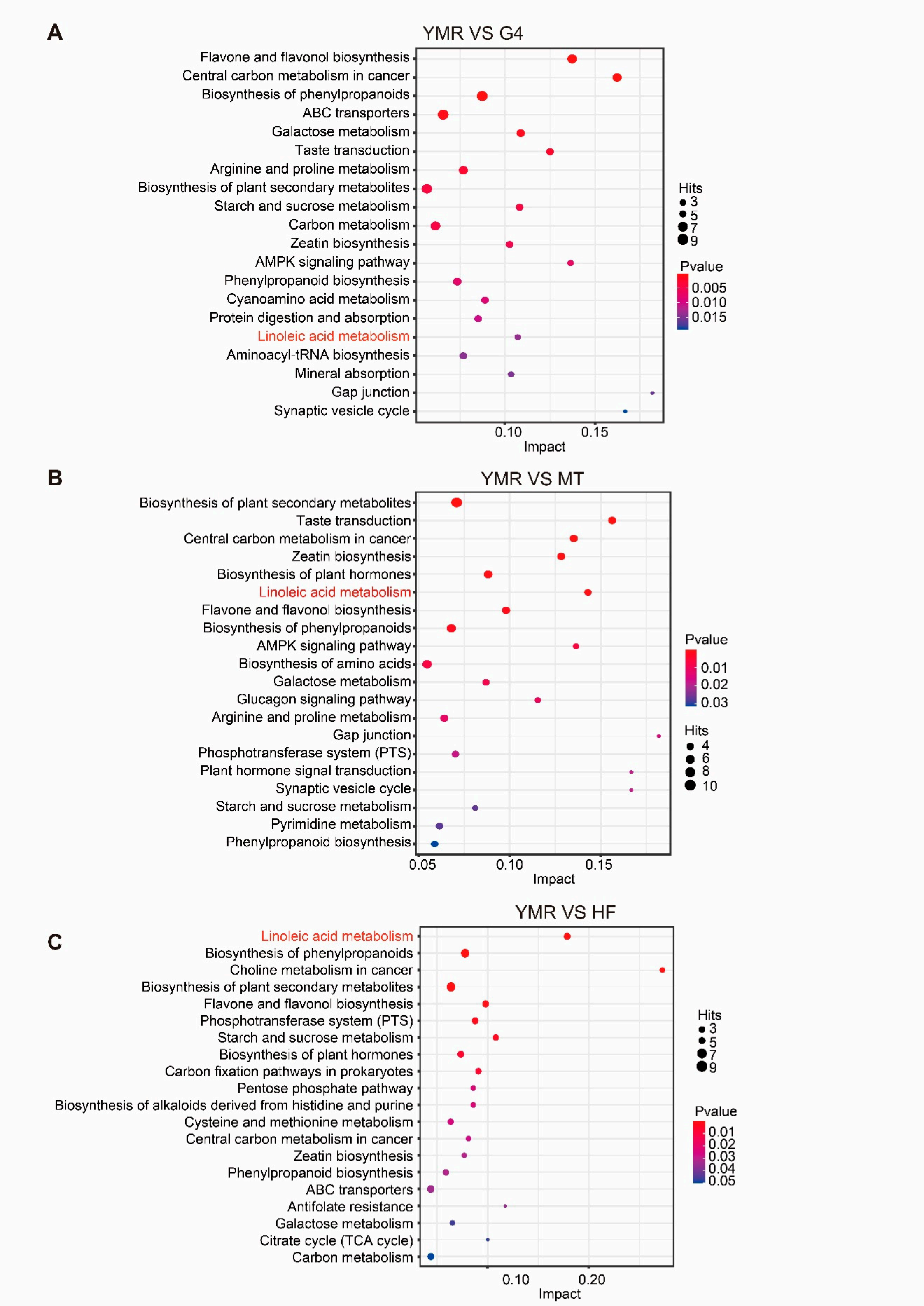

Figure S4. KEGG pathway enrichment analysis of differentially expressed metabolites (DEMs) across four *M. laevigata*. (A) Pathway enrichment profiles of

DEMs identified in YMR versus HF fruits; (B) Pathway enrichment profiles of DEMs identified in YMR versus MT fruits; (C) Pathway enrichment profiles of DEMs identified in YMR versus G4 fruits.

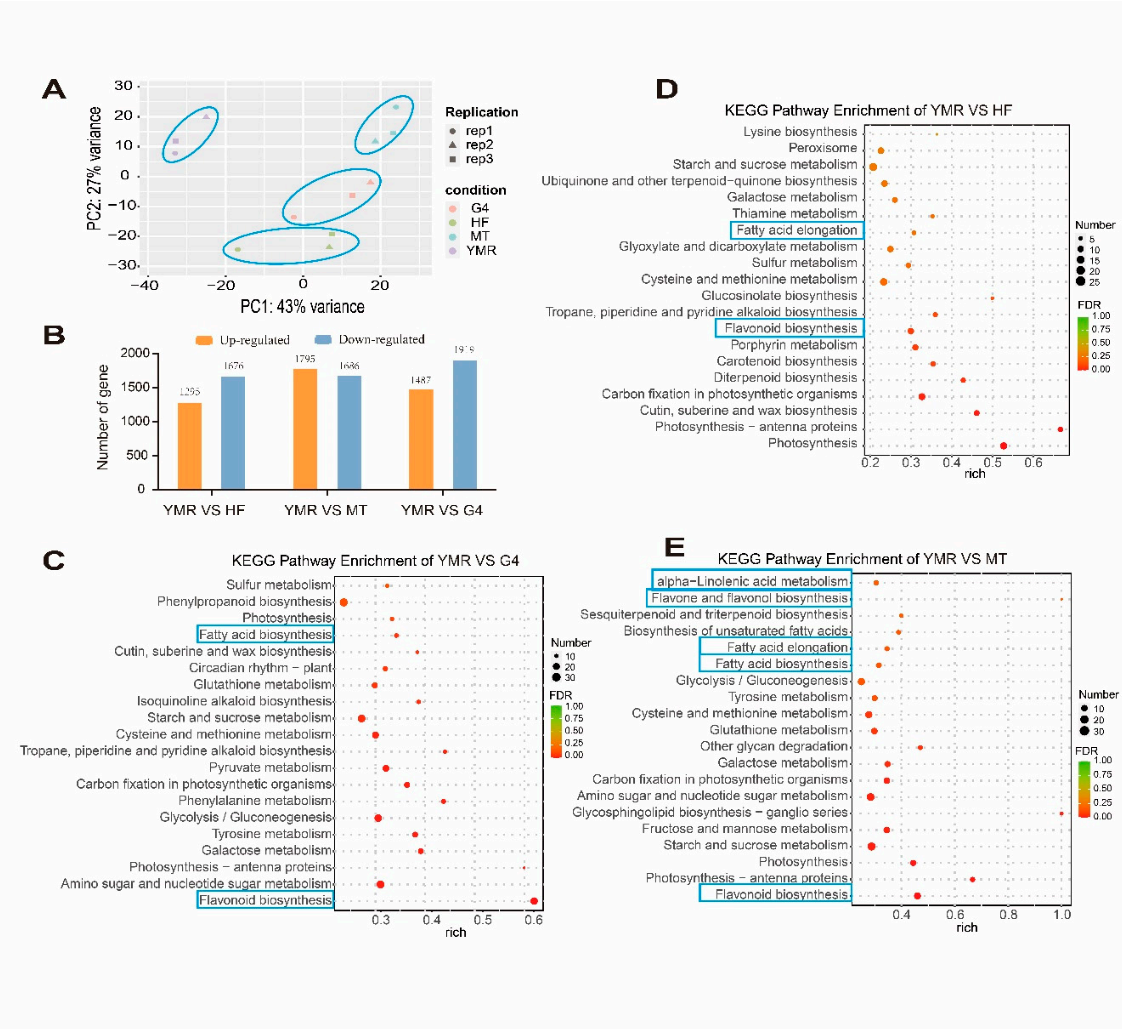

Figure S5. Comprehensive transcriptomic analysis and differential gene expression profiling across four *M. laevigata*. (A) Principal component analysis of global gene expression patterns; (B) Distribution of differentially expressed genes across comparison groups; (C) KEGG pathway enrichment analysis of differentially expressed genes (DEGs) in YMR versus HF fruits; (D) KEGG pathway enrichment analysis of DEGs in YMR versus MT fruits; (E) KEGG pathway enrichment analysis of DEGs in YMR versus G4 fruits.
